# Exosomal miR‐95‐5p regulates chondrogenesis and cartilage degradation via histone deacetylase 2/8

**DOI:** 10.1111/jcmm.13808

**Published:** 2018-07-31

**Authors:** Guping Mao, Shu Hu, Ziji Zhang, Peihui Wu, Xiaoyi Zhao, Ruifu Lin, Weiming Liao, Yan Kang

**Affiliations:** ^1^ Department of Joint Surgery First Affiliated Hospital of Sun Yat‐sen University Guangzhou Guangdong China

**Keywords:** exosomes, HDAC2, HDAC8, miRNA‐95‐5p, osteoarthritis

## Abstract

MicroRNAs play critical roles in the pathogenesis of osteoarthritis, the most common chronic degenerative joint disease. Exosomes derived from miR‐95‐5p‐overexpressing primary chondrocytes (AC‐miR‐95‐5p) may be effective in treating osteoarthritis. Increased expression of HDAC2/8 occurs in the tissues and chondrocyte‐secreted exosomes of patients with osteoarthritis and mediates cartilage‐specific gene expression in chondrocytes. We have been suggested that exosomes derived from AC‐miR‐95‐5p (AC‐miR‐95‐5p‐Exos) would enhance chondrogenesis and prevent the development of osteoarthritis by directly targeting HDAC2/8. Our in vitro experiments showed that miR‐95‐5p expression was significantly lower in osteoarthritic chondrocyte‐secreted exosomes than in normal cartilage. Treatment with AC‐miR‐95‐5p‐Exos promoted cartilage development and cartilage matrix expression in mesenchymal stem cells induced to undergo chondrogenesis and chondrocytes, respectively. In contrast, co‐culture with exosomes derived from chondrocytes transfected with an antisense inhibitor of miR‐95‐5p (AC‐anti‐miR‐95‐5p‐Exos) prevented chondrogenic differentiation and reduced cartilage matrix synthesis by enhancing the expression of HDAC2/8. MiR‐95‐5p suppressed the activity of reporter constructs containing the 3ʹ‐untranslated region of *HDAC2/8*, inhibited HDAC2/8 expression and promoted cartilage matrix expression. Our results suggest that AC‐miR‐95‐5p‐Exos regulate cartilage development and homoeostasis by directly targeting HDAC2/8. Thus, AC‐miR‐95‐5p‐Exos may act as an HDAC2/8 inhibitor and exhibit potential as a disease‐modifying osteoarthritis drug.

## 1 INTRODUCTION

Osteoarthritis (OA) is the most common form of arthritis and represents a major socio‐economic burden,^1^, ^2^ which is characterized as articular cartilage destruction and impaired function of the affected joints, and is a main cause of pain and disability^3^. More and more evidence showed that epigenetic modification plays a vital role in chondrocytes growth and development and the initiation and progression of OA. These include chemical modifications to DNA, post‐translational modifications to histone tails, and non‐coding RNAs that contribute to cellular changes.^4^, ^5^, ^6^, ^7^, ^8^ It is widely accepted that the activation of histone deacetylase (HDAC) plays a pivotal role in the initiation and progression of OA.^9^, ^10^, ^11^, ^12^, ^13^ For example, HDAC4 as a central regulator of chondrocyte hypertrophy and skeletogenesis, which directly inhibited the expression of Runt‐related transcription factor 2 (RUNX2).^14^, ^15^ In contrast, HDAC1, 2, 3 and 8 tend to impede cartilage development by inhibiting the expression of cartilage‐specific genes, such as *COL2A1* and aggrecan.^16^, ^17^, ^18^ Moreover, the inhibition of HDAC1/2/3 represses interleukin (IL)‐1‐induced matrix metalloproteinase (MMP) expression and cartilage resorption.^9^

Exosomes are packaged vesicles 50‐150 nm in diameter, which contain specific proteins, lipids and/or nucleic acids (DNA, mRNAs, miRNAs and other non‐coding RNAs). They are secreted by almost all metabolically active cells and exert their functions in recipient cells.^19^, ^20^, ^21^, ^22^ Exosomes are cellular by‐products that reflect the pathophysiological changes occurring nearby or in the surrounding environment, making them key factors in monitoring disease initiation and progression.^23^

MicroRNAs (miRNAs) are small non‐coding RNAs that function as post‐transcriptional regulators. They silence the 3′‐untranslated regions (3′‐UTRs) of target genes and suppress their expression^24^. Recent studies have indicated significant regulatory roles of miRNAs and HDACs in chondrogenesis and cartilage degeneration. For example, miR‐92a‐3p regulates the expression of cartilage‐specific genes by directly targeting HDAC2 in chondrogenesis and cartilage degradation,^17^ and miR‐455‐3p modulates cartilage development and degeneration through HDAC2/8.^18^ Furthermore, miRNA‐222 modulates MMP‐13 expression by inhibiting HDAC4 during the initiation and progression of OA,^25^ and miR‐140 and miR‐381 target HDAC4 to regulate cartilage and bone formation.^26^, ^27^ HDAC inhibitors (HDACi) up‐regulate the level of miRNA‐146a and repress IL‐1β‐induced signalling and cytokine secretion in OA fibroblast‐like synoviocytes.^28^ However, changes in the exosomal miRNA content associated with cartilage development and degeneration have not yet been described. The primary goal of this study was to illustrate differences in exosomal miRNAs in normal and OA cartilage‐secreted exosomes and to explore their biological functions.

In this study, we used a miRNA microarray to analyse the miRNA profiles of normal and OA cartilage‐secreted exosomes and observed the down‐regulation of miR‐95‐5p in OA. We found that miR‐95‐5p has the potential to regulate *HDAC2/8* expression with miRNA target prediction software. However, no previous studies on the function of miR‐95‐5p have been conducted. Given the role of exosomal miRNAs in regulating cartilage homoeostasis and chondrogenesis, we hypothesized that exosomal miR‐95‐5p may play a role in both cartilage development and OA pathogenesis. In this study, we aimed to determine whether exosomal miR‐95‐5p regulates cartilage‐specific gene expression by targeting *HDAC2/8* in chondrogenesis and cartilage degradation.

## 2 MATERIALS AND METHODS

### 2.1 Ethics

All procedures were approved by the ethical committee of the First Affiliated Hospital of SunYat‐Sen University (IRB: 2014C‐028) and the Declaration of Helsinki (2000). All volunteers provided written informed consent.

### 2.2 Culture of human mesenchymal stem cells and chondrogenesis in micromass culture

Bone marrow samples were obtained by iliac crest aspiration from 6 normal human donors (mean age: 35 years; range: 31‐39 years; male: 3, female: 3). The isolation of human mesenchymal stem cells (hMSCs) was carried out using a density gradient centrifugation method.^17^ Cells were cultured in basal medium (alpha‐modified Eagle's medium [α‐MEM]; Gibco Life Technology, Grand Island, NY, USA) supplemented with 10% foetal bovine serum (FBS; Gibco Life Technology) and 1% penicillin/streptomycin (Gibco Life Technology). Cells were cultured at 37°C under a 5% CO_2_ atmosphere, and the medium was changed every 3 days. The hMSCs were induced to differentiate into chondrocytes in micromass culture within three passages as described previously.^17^

### 2.3 Isolation and identification of exosomes

Exosome isolation was carried out using ultracentrifugation.^29^ In brief, chondrocytes culture supernatants were subjected to successive centrifugations at 3000 *g* (30 minutes) and 10 000 *g* (30 minutes). Exosomes were then pelleted at 64 000 *g* for 110 minutes using an SW28 rotor (Beckman Coulter, California, USA). Exosome pellets were resuspended in 0.32 mol/L sucrose and centrifuged at 100 000 *g* for 1 hour (SW60Ti rotor; Beckman Coulter). The exosome pellet was then resuspended in phosphate‐buffered saline (PBS). Nanosight 2000 analysis and transmission electron microscopy (TEM) were used to identify exosomes. RNA and proteins were extracted from exosomes using a Total Exosome RNA & Protein Isolation Kit (Invitrogen, Carlsbad, CA, USA) for further analysis. The BCA protein assay kit was used to quantify the exosomes.

### 2.4 Primary chondrocyte collection, isolation and cell culture

Degraded joint cartilage samples were obtained from patients (n = 6; mean ± standard deviation [SD] age: 58.2 ± 1.24 years; male: 3, female: 3) with OA knee joints during total knee replacement operations. Normal cartilage samples were taken from patients (n = 6; mean ± SD age: 53.6 ± 1.48 years; male: 3, female: 3) with no previous history of OA or rheumatoid arthritis, who underwent total hip replacement surgery because of fractures of the femoral neck. The cartilages were dissected away from the subchondral bone and then digested by 4 mg/mL protease and 0.25 mg/mL collagenase P as described previously.^17^ Cells were cultured in DMEM/Nutrient Mixture F‐12 (Gibco Life Technology) containing 5% foetal bovine serum (FBS; Gibco Life Technology), 100 IU/mL penicillin and 100 μg/mL streptomycin (Gibco Life Technology). The chondrocytes were used in experiments within 3‐7 days and without passaging to avoid dedifferentiation.

### 2.5 Chondrocyte proliferation assay

The effect of AC‐Exos and AC‐miR‐95‐5p‐Exos on the proliferation of human chondrocytes was evaluated using the Cell Counting Kit‐8 (CCK‐8; Dojindo, Kyushu Island, Japan) as described previously.^30^ Chondrocytes were seeded into 96‐well plates at 2 × 10^3^ cells/well. After 12 hours, different doses of AC‐Exos or AC‐miR‐95‐5p‐Exos were added to the wells. Cell proliferation curves were constructed by measuring the amount of formazan dye generated by cellular dehydrogenase activity with a microplate reader at a wavelength of 450 nm.

### 2.6 RNA extraction, reverse transcription and quantitative real‐time polymerase chain reaction (qRT‐PCR)

RNA extraction and reverse transcription were performed as described previously.^17^ CDNA was generated using the PrimeScript miRNA cDNA Synthesis Kit (Takara Biotechnology, Shiga, Japan) following the manufacturer's instruction. Transcript levels were normalized to that of the housekeeping gene glyceraldehyde 3‐phosphate dehydrogenase (GAPDH; for mRNA) or the small U6 RNA (for miRNA). The specific primers used for these analyses are listed in Table 1. Gene expression was calculated using the 2^−ΔΔCt^ method, and each experiment was performed in triplicate.

**Table 1:** Primers for quantitative real‐time polymerase chain reaction (qRT‐PCR)

Gene		Primer sequence(5′‐3′)
hsa‐COL2A1	F	GCACCTGCAGAGACCTGAAAC
hsa‐COL2A1	R	GCAAGTCTCGCCAGTCTCCA
hsa‐COL9A1	F	GGCAGTAGAGGAGAATTAGGACC
hsa‐COL9A1	R	GTTCACCGACTACACCCCTG
hsa‐COL10A1	F	CATAAAAGGCCCACTACCCAAC
hsa‐COL10A1	R	ACCTTGCTCTCCTCTTACTGC
hsa‐SOX9	F	GGAGATGAAATCTGTTCTGGGAATG
hsa‐SOX9	R	TTGAAGGTTAACTGCTGGTGTTCTG
hsa‐RUNX2	F	CACTGGCGCTGCAACAAGA
hsa‐RUNX2	R	CATTCCGGAGCTCAGCAGAATAA
hsa‐HDAC2	F	ACCCACCCCGACTTAACAAA
hsa‐HDAC2	R	GAGGGAGGATTACAAACGACAAAG
hsa‐HDAC8	F	TCGCTGGTCCCGGTTTATATC
hsa‐HDAC8	R	TACTGGCCCGTTTGGGGAT
hsa‐COMP	F	GATCACGTTCCTGAAAAACACG
hsa‐COMP	R	GCTCTCCGTCTGGATGCAG
hsa‐ACAN	F	GATGTTCCCTGCAATTACCACCTC
hsa‐ACAN	R	TGATCTCATACCGGTCCTTCTTCTG
hsa‐MMP‐13	F	TCCTGATGTGGGTGAATACAATG
hsa‐MMP‐13	R	GCCATCGTGAAGTCTGGTAAAAT
hsa‐GAPDH	F	GCACCGTCAAGGCTGAGAAC
hsa‐GAPDH	R	TGGTGAAGACGCCAGTGGA
hsa‐U6	F	CTCGCTTCGGCAGCACA
hsa‐U6	R	AACGCTTCACGAATTTGCGT
hsa‐miR‐95‐5p	F	TCAATAAATGTCTGTTGAATT
hsa‐miR‐665	F	GTCGTATCCAGTGCAGGGTCCGAGGTATTCGCACTGGATACGACAGGGGC
hsa‐491‐3p	F	CTTATGCAAGATTCCCTTCTAC
hsa‐382‐3p	F	AATCATTCACGGACAACACTT
hsa‐345‐3p	F	GCCCTGAACGAGGGGTCTGGAG
hsa‐4678	F	GTTCAGACTTATGATTTTTGGGGTCAAAGATTCTGAGCAATAACCTATTAAA
hsa‐660‐5p	F	TACCCATTGCATATCGGAGTTG
hsa‐199b‐5p	F	CCCAGTGTTTAGACTATCTGTTC

### 2.7 Microarray analysis

The miRNA expression profiling assays from six samples exosomes (three paired comparison, with and without osteoarthritis from three donors) were conducted by the use of Exiqon miRCURY™ LNA arrays (v18.0; contains 2043 capture probes covering all human miRNAs; Exiqon, Vedbaek, Denmark). Microarray experiments and data analyses were performed by KangChen Bio‐tech, Shanghai, China.

### 2.8 Transfection

The normal chondrocytes were transfected with miR‐95‐3p mimic or inhibitor (RiboBio, Guangzhou, China) at a concentration of 50 nmol/L; they were also transfected with HDAC2/8 siRNA or NC (RiboBio). Lipofectamine® 2000 Transfection Reagent (Gibco Life Technologies) was used to transfect cells according to the manufacturer's instructions. Cells were then harvested after 48 hours for quantitative real‐time reverse transcription‐polymerase chain reaction (qRT‐PCR), or after 72 hours for western blot analysis.

### 2.9 Western blot analysis and immunohistochemical analysis

Western blot analysis was carried out as described previously.^17^ Briefly, total proteins were isolated from normal or OA chondrocytes by RIPA buffer (Beyotime Biotechnology, Beijing, China) containing protease inhibitors (Abcam, Cambridge, UK) to obtain whole cell extracts. Membranes were incubated with primary antibodies against RUNX2 (1:1000 dilution; Cell Signalling Technology, Boston, USA); β‐actin (1:3000; Cell Signalling Technology); HDAC2, HDAC8, COL2A1 and MMP13 (1:1000; Abcam); aggrecan and SOX9 (1:2000; Millipore, Bedford, USA). β‐actin was used as an internal control. Immunohistochemical analysis was performed as described previously.^17^ Cartilage tissue sections were blocked in PBS plus 0.025% Tween 20 with 10% foetal bovine serum, followed by incubation with rabbit anti‐human HDAC2/8 antibody (1:200; Abcam).

### 2.10 Luciferase constructs and reporter assay

The 3′‐UTR fragments of the HDAC2, and HDAC8 coding sequences were PCR‐amplified using the following primers: hsa‐HDAC2‐3′‐UTR‐F, ATAGGCCGGCATAGACGCGTATTTGACAGTCTCACCAATTTCAGAAAATC, hsa‐HDAC2‐3′‐UTR‐R, AAAGATCCTTTATTAAGCTTAGTTACCCCCATTTGTCTGCTTCCTGC; hsa‐HDAC8‐3′‐UTR‐F, ATAGGCCGGCATAGACGCGTAAGCAGCTGCCGCAGCTTGTCTTCC, hsa‐HDAC8‐3′‐UTR‐R, AAAGATCCTTTATTAAGCTTTAACATTTATTGAGAACTTTATTCAGAATAGA. Meanwhile, the following primers were used for the mutated (mut) sequences: hsa‐HDAC2‐mut‐F, CTTCATAATTAGAAAAACTCG**TTTCGTG**ACTTGCCAAGAAAAACAAAAACG, hsa‐HDAC2‐mut‐R, CGTTTTTGTTTTTCTTGGCAAGT**CACGAAA**CGAGTTTTTCTAATTATGAAG; hsa‐HDAC8‐mut‐F, CAATACTTTATACATAGTAGGCATA**TTTCGTG**TAGTCATTAAATGAATACATG, hsa‐HDAC8‐mut‐R, CATGTATTCATTTAATGACTA**CACGAAA**TATGCCTACTATG TATAAAGTATTG; For reporter assay analyses, 1.2 × 104 cells (HEK293T) in a 96‐well plate were transfected with 50 nM hsa‐miR‐95‐5p or mimic NC (RiboBio). The cells were then co‐transfected with 2 μg/mL of vector with the wild‐type or mutant HDAC2/8 3′‐UTR. Luciferase activity was measured 48 hours, after transfection by the Dual‐Luciferase® Reporter Assay System (Promega, Madison, WI, USA) according to the manufacturer's instructions. Luciferase assays were performed in three independent experiments.

### 2.11 Statistical analysis

Data are presented as means ± standard deviations (SD) of the results of at least three independent experiments. Student's *t* tests and Mann‐Whitney *U* tests were used to identify differences between groups as appropriate. One‐way analysis of variance (ANOVA) and Kruskal‐Wallis tests were carried out for multiple group comparisons. A *P*‐value <0.05 was considered statistically significant.^31^ All analyses were performed using SPSS Version 20 (IBM Corporation, Armonk, NY, USA).

## 3 RESULTS

### 3.1 Identification of exosomes derived from chondrocytes

**Figure 1:**
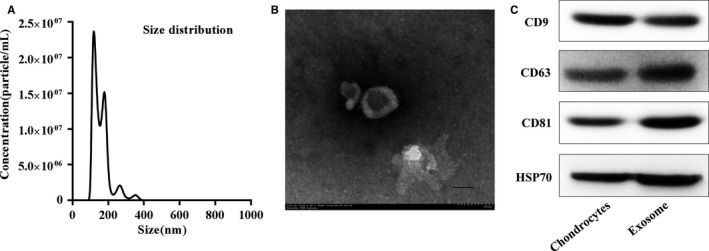
Characterization of chondrocytes‐derived exosomes. A, Particle size distribution of exosomes measured by Nanosight. B, Morphology of exosomes observed by transmission electron microscopy (TEM). Scale bar 100 nm. C, Exosome surface markers (CD9, CD63, CD81, and Hsp70) measured using western blotting. The cartilage exosomes were obtained by chondrocytes culture supernatants via ultracentrifugation. This experiment was repeated independently three times and representative results are shown

Nanosight analysis showed that the size of the majority of chondrocyte‐secreted exosomes was approximately 120 ± 30 nm (Figure 1A). TEM clearly revealed that chondrocyte‐secreted exosomes exhibited a cup‐shaped or round morphology (Figure 1B). Western blotting analyses confirmed that chondrocyte‐secreted exosomes expressed exosomal markers, such as CD9, CD63, CD81 and HSP70 (Figure 1C).

### 3.2 MiRNA expression profiles in OA and normal chondrocyte‐secreted exosomes

**Figure 2:**
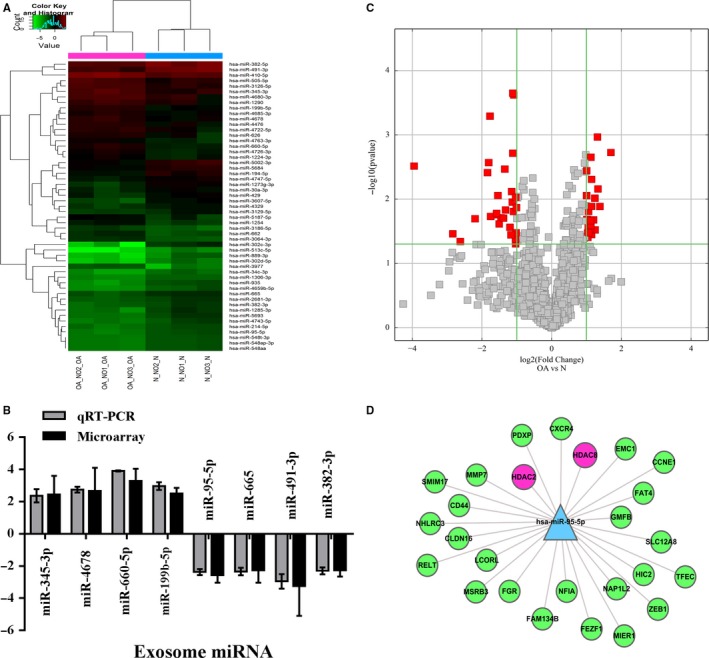
Exosomal miRNA expression profiles in chondrocyte‐derived exosomes. A, Hierarchical Clustering shows a distinguishable exosomal miRNA expression profile between the two groups and homogeneity within groups. RNA was extracted from chondrocytes‐derived exosomes samples obtained from three OA patients and three control subjects. B, qRT‐PCR confirmation of miRNA expression in OA chondrocyte‐secreted exosomes and normal chondrocyte‐secreted exosomes. Quantitative real‐time PCR was performed with indicated primers and fold‐change of mRNA was normalized to U6 mRNA. Samples from three OA or normal chondrocytes and each were repeated three times. C, Volcano Plot of the differentially expressed exosomal miRNA. The red points in the plot represent the differentially expressed exosomal miRNA with statistical significance. D, Some of target gene of miR‐95‐5p, which can directly target HDAC2 and HDAC8

The expression profiles of miRNAs in OA and normal chondrocyte‐secreted exosomes were detected using a miRNA microarray. SAM statistical software was used to identify differentially expressed miRNAs between OA‐secreted exosomes and normal chondrocyte‐secreted exosomes. The differentially expressed miRNAs from all three paired samples are shown in Figure 2A‐C and Table S1. Among the miRNAs that were consistently differentially expressed in all three paired samples, 22 were up‐regulated (fold change >2) and 29 were down‐regulated (fold change <−2) in OA‐secreted exosomes compared to levels in normal chondrocyte‐secreted exosomes, as shown in Table S1. Exosomal miR‐95‐5p was down‐regulated 2.54‐fold in OA‐secreted exosomes. We predicted the target genes of miR‐95‐5p with TargetScan and microRNA.org and identified *HDAC2* and *HDAC8* (Figure 2D). Previous studies have demonstrated that *HDAC2/8* are closely related to the initiation and development of OA.^16^, ^17^, ^18^ These data indicate that exosomal miR‐95‐5p plays an important role in maintaining cartilage stability.

### 3.3 Expression levels of exosomal miR‐95‐5p and HDAC2/8 in normal and OA cartilage

**Figure 3:**
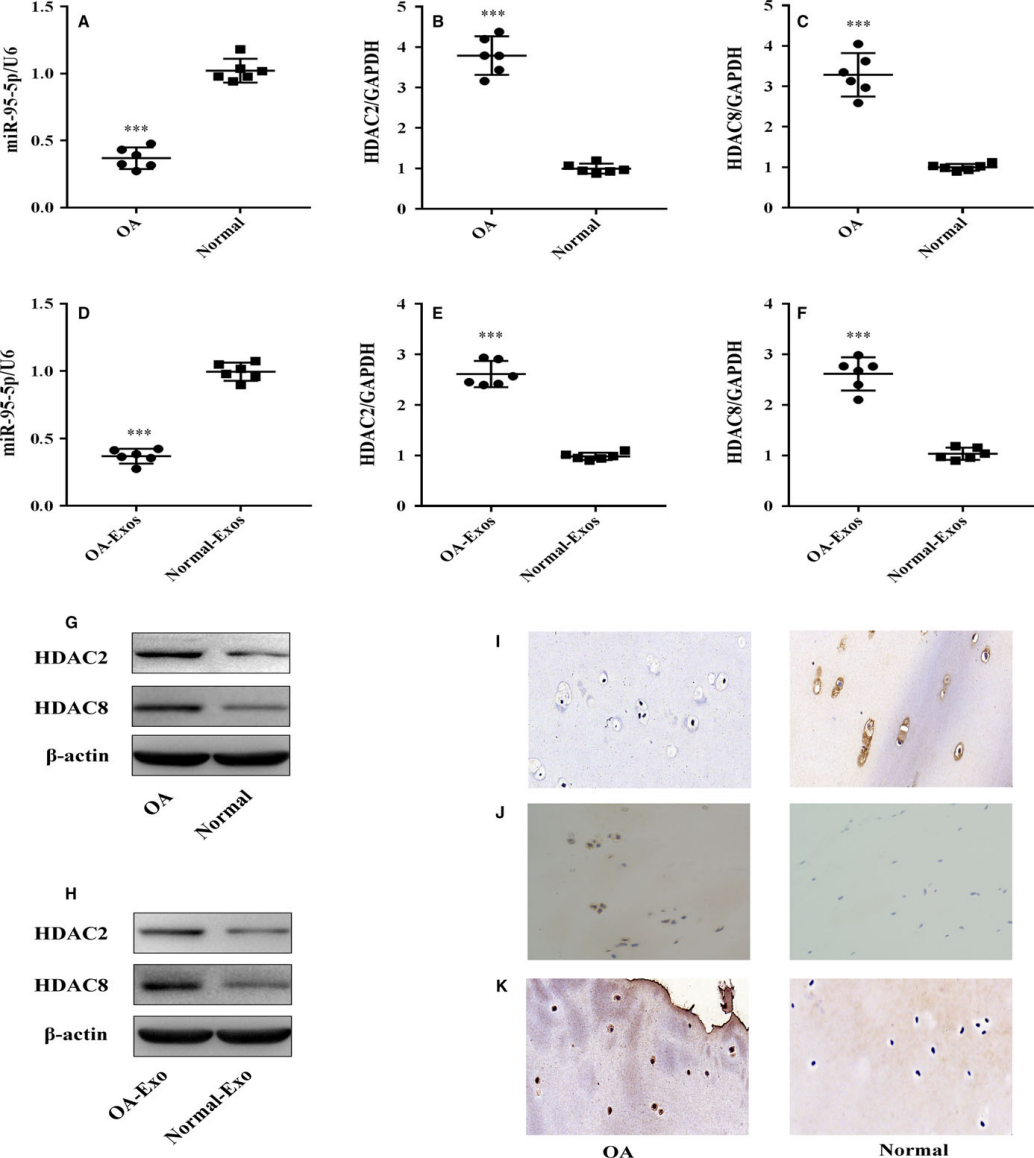
The expression of mature miR‐95‐5p and HDAC2/8 in normal and OA cartilage. A‐C, Relative miR‐95‐5p and HDAC2/8 mRNA levels in normal and OA cartilage tissues and (D‐F) in normal and OA cartilage‐secreted exosome were determined by SYBR green‐based qRT‐PCR. U6 and GAPDH were used as endogenous controls. Each dot represents a value from a single experiment of one donor. The bar shows the mean and 95% confidence intervals of the values from six different donors per group. ***P < 0.001. G, H, J, K, HDAC2/8 protein levels in normal cartilage and OA tissues or cartilage‐secreted exosomes were determined by Western blotting and immunohistochemistry using anti‐HDAC2/HDAC8 monoclonal antibody and β‐actin as endogenous controls. Data shown are representative of results from six normal and OA cartilages (magnification, ×200). I, The miR‐95‐5p expression levels were determined in normal cartilage and OA cartilage by in situ hybridization (magnification, ×200). Scale bar 50 μm

To investigate whether the expression of exosomal miR‐95‐5p changes during the progression of OA, we showed the miR‐95‐5p expression in normal and OA cartilage‐secreted exosomes. The expression of exosomal miR‐95‐5p was markedly reduced in OA cartilage compared to levels in normal cartilage (Figure 3A,D, qRT‐PCR analysis; Figure 3I, in situ hybridization). However, the higher mRNA and protein levels of HDAC2/8 were exhibited in OA cartilage compared to levels in normal cartilage (Figure 3B,C,E,F, qRT‐PCR analysis; Figure 3G,H, western blot; Figure 3J,K, immunohistochemical analysis).

### 3.4 Exosomal miR‐95‐5p enhances histone H3 acetylation and maintains the function of articular chondrocytes

**Figure 4:**
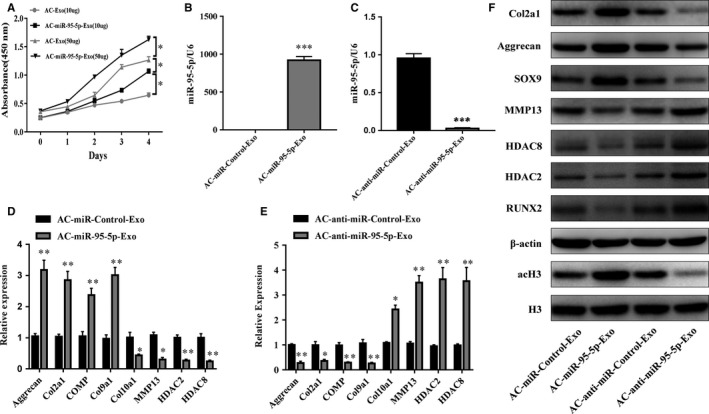
Responses of articular chondrocytes to stimulation by AC‐ miR‐95‐5p‐Exos. A, AC‐Exos and ACmiR‐95‐5p‐Exos co‐cultured with chondrocytes at different doses. B, C, Expression level changes of miR‐95‐5p in AC‐miR‐Control‐Exos and AC‐miR‐95‐5p‐Exos, or AC‐anti‐miR‐Control‐Exos and AC‐anti‐miR‐95‐5p‐Exos were determined by SYBR green‐based qRT‐PCR. U6 was used as endogenous controls. Chondrocytes were treated with AC‐miR‐Control‐Exos and AC‐miR‐95‐5p‐Exos or AC‐anti‐miR‐Control‐Exos and AC‐antimiR‐95‐5p‐Exos. D, E, The gene expression levels of aggrecan, Col2a1, Col9a1, COMP, Col10a1, MMP13, HDAC2 and HDAC8 were estimated by qRT‐PCR. Quantitative data are presented as means ± standard deviations from three independent experiments. GAPDH was used as internal controls for mRNA. *P < 0.05, **P < 0.01, ***P < 0.001. F, The acetylation level of histone H3 and expression levels of aggrecan, Col2a1, SOX9, RUNX2, MMP13, HDAC2 and HDAC8 were visualized by Western blotting and β‐actin and total histone H3 were internal controls as endogenous control

Proliferation was evaluated following the stimulation of chondrocytes with various doses of AC‐Exos or AC‐miR‐95‐5p‐Exos. At a concentration of 50 μg exosomes/mL, chondrocytes cultured with AC‐miR‐95‐5p‐Exos showed greater proliferation than those incubated with other doses of exosomes (Figure 4A). In order to determine the effect of exosomal miR‐95‐5p on chondrocyte function, we treated chondrocytes with 50 μg/mL AC‐miR‐95‐5p‐Exos or AC‐anti‐miR‐95‐5p‐Exos. We found that AC‐miR‐95‐5p‐Exos significantly up‐regulated the mRNA expression levels of aggrecan, *COL2A1*,* COL9A1* and *COMP* and decreased the expression levels of *COL10A1*,* MMP13*,* HDAC2* and *HDAC8* (Figure 4D). In contrast, AC‐anti‐miR‐95‐5p‐Exos accelerated cartilage matrix degradation and increased *HDAC2/8* expression (Figure 4E). Moreover, AC‐miR‐95‐5p‐Exos decreased HDAC2 and HDAC8 protein expression while increasing the protein expression of acetylated histone H3 (AcH3), COL2A1, aggrecan and SOX9, as determined by western blotting (Figure 4F). Consistent with these findings, treated with AC‐anti‐miR‐95‐5p‐Exos up‐regulated HDAC2 and HDAC8 protein expression in PHCs (Figure 4F). These data indicate that AC‐miR‐95‐5p‐Exos may slow the progression of OA and maintain cartilage development and homoeostasis.

### 3.5 Exosomal miR‐95‐5p regulates cartilage development in hMSCs during chondrogenesis

**Figure 5:**
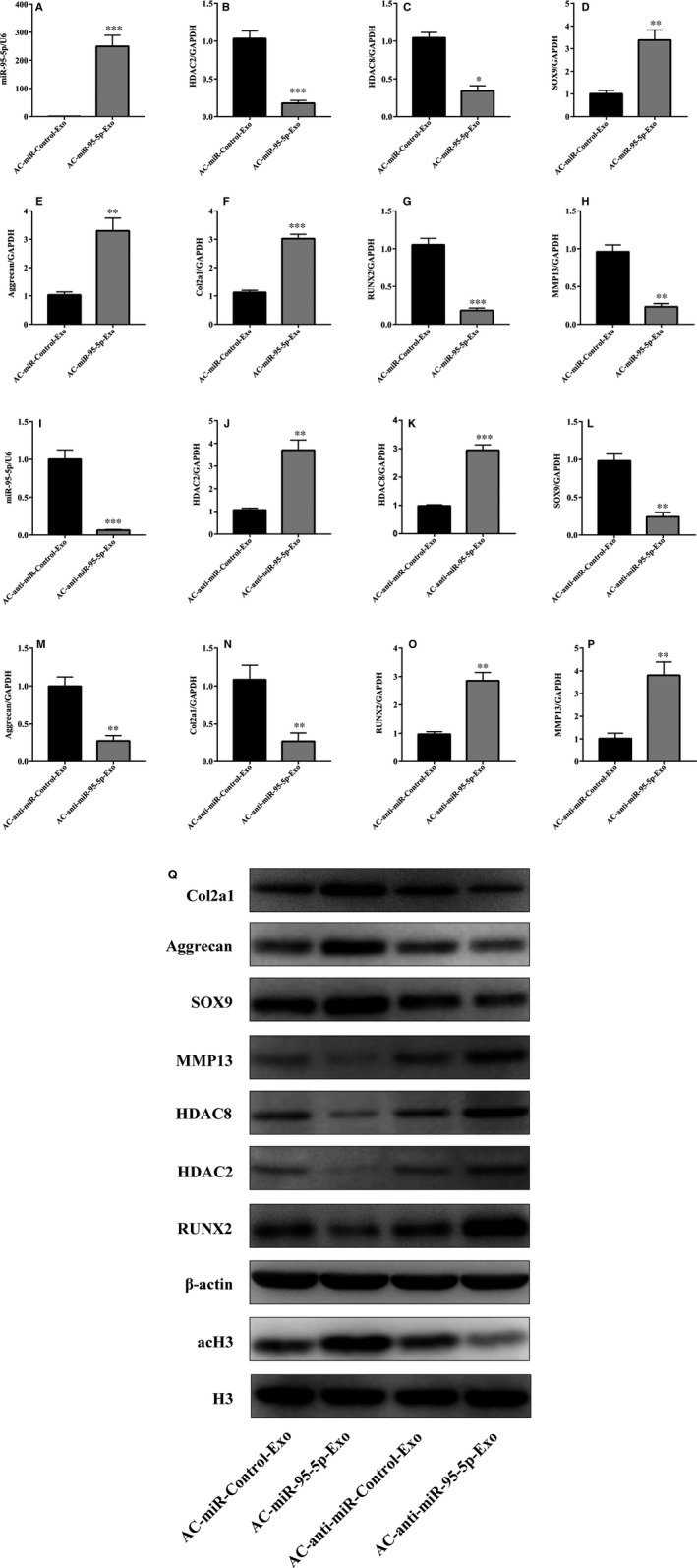
AC‐miR‐95‐5p‐Exos regulates the expression of HDAC2/8, Col2A1, aggrecan and SOX9 during chondrogenesis. HMSCs were treated with TGF‐β3 to induce chondrogenesis and co‐cultured with AC‐miR‐95‐5p‐Exos or AC‐anti‐miR‐95‐5p‐Exos. (A, I) The expression level of miR‐95‐5p were estimated by qRTPCR, while the expression levels of HDAC2 (B, J, Q), HDAC8 (C, K, Q), SOX9 (D, L, Q), Aggrecan (E, M), COL2A1 (F, N, Q), RUNX2 (G, O, Q) and MMP13 (H, P, Q) were estimated by both qRT‐PCR and western blotting. U6, GAPDH and β‐actin were used as endogenous controls. Data were presented as means ± standard deviation of three samples. *P < 0.05, **P < 0.01, ***P < 0.001

To further determine whether exosomal miR‐95‐5p regulates the chondrogenesis of hMSCs, we overexpressed or inhibited miR‐95‐5p in human primary chondrocytes. Chondrocytes were transfected with miR‐95‐5p or its antisense inhibitor (anti‐miR‐95‐5p), and exosomes were harvested (AC‐miR‐95‐5p‐Exos or AC‐anti‐miR‐95‐5p‐Exos, respectively). Next, hMSCs were induced to differentiate into chondrocytes and were treated with 50 μg/mL AC‐miR‐95‐5p‐Exos or AC‐anti‐miR‐95‐5p‐Exos in micromass culture for 14 days (Figure 5). The mRNA and protein expression levels of aggrecan, COL2A1 and SOX9 (Figure 5D‐F) were significantly up‐regulated and those of HDAC2, HDAC8, RUNX2 and MMP13 (Figure 3B,C,G,H) were significantly down‐regulated in the AC‐miR‐95‐5p‐Exos co‐cultures, as determined by qPCR and western blotting (Figure 5Q). In contrast, HDAC2, HDAC8, RUNX2 and MMP13 levels were significantly up‐regulated (Figure 5J,K,O,P,Q) and COL2A1, aggrecan and SOX9 were significantly down‐regulated (Figure 5L,M,N,Q) in AC‐anti‐miR‐95‐5p‐Exos co‐cultures. These data indicate that miR‐95‐5p may target *HDAC2/8* to promote SOX9, COL2A1 and aggrecan expression and enhance cartilage development.

### 3.6 SiHDAC2/8 promote cartilage‐specific gene expression

**Figure 6:**
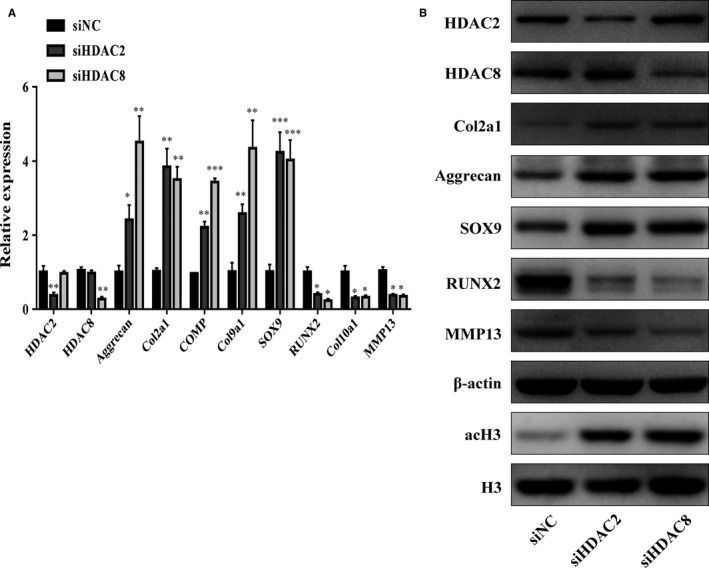
PHCs were transfected with siNC (as control) or siHDAC2/8. The gene expression levels of aggrecan, Col2a1, COMP, Col9a1, Col10a1, SOX9, RUNX2, MMP13, HDAC2 and HDAC8 were estimated by qRT‐PCR (A). Quantitative data are presented as means ± standard deviations from three independent experiments. GAPDH was used as internal controls for mRNA, *P < 0.05, **P < 0.01, ***P < 0.001. The acetylation of histone H3 and expression of HDAC2, HDAC8, SOX9, RUNX2, MMP13, Col2a1 and aggrecan were visualized by Western blot and β‐actin and total histone H3 were internal controls as endogenous control

To perform the converse of the above experiments, a siRNA approach was used to reduce the levels of HDAC2/8. Chondrocytes were transfected with siHDAC2/8, resulting in the down‐regulation of *HDAC2/8* and other cartilage‐related gene levels, as shown by qRT‐PCR (Figure 6A). This reduction was correlated with a decrease in cartilage HDAC2/8 protein expression, as well as increases in the expression of COL2A1, aggrecan and SOX9, as shown in Figure 6B. These data indicate that the down‐regulation of HDAC2/8 promotes increases in the expression of cartilage‐specific proteins.

### 3.7 MiR‐95‐5p directly targets the 3ʹ‐UTRs of *HDAC2/8* mRNA

**Figure 7:**
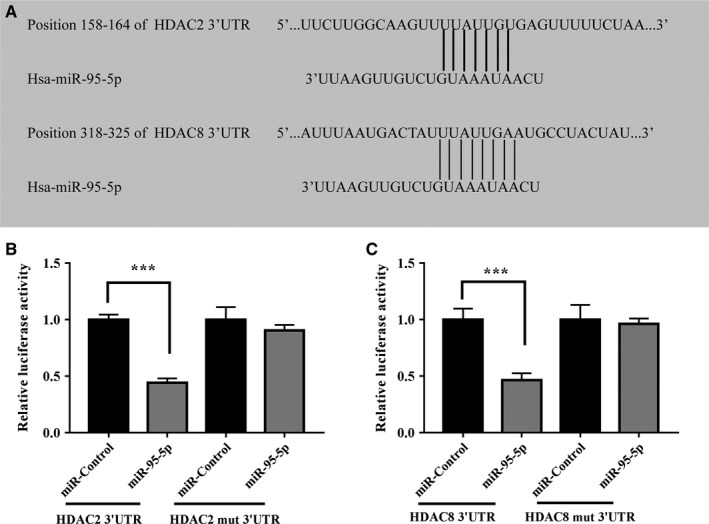
MiR‐95‐5p directly targets HDAC2/8. HDAC2/8 were predicted potential targets of miR‐95‐5p. Alignment of HDAC2/8 3′‐UTR miR‐95‐5p were shown (A). A luciferase reporter carrying the 3′‐UTR of HDAC2/8 or mutant HDAC2/8 in which the binding site of miR‐95‐5p were mutated (Luc‐HDAC2/8‐UTR‐mut) was introduced into 293T cells along with negative miR‐control (NC), miR‐95‐5p. The cells were harvested 48 h later for luciferase assays (B, C). Quantitative data are presented as means ± standard deviations from three independent experiments, ***P < 0.001

To further clarify the molecular mechanisms that underlie the regulation of *HDAC2/8* expression by miR‐95‐5p, we analysed the sequences of the 3ʹ‐UTRs of the human *HDAC2/8* genes. Bioinformatics software such as TargetScan (http://www.targetscan.org) and miRanda (http://www.microrna.org) revealed that the 3ʹ‐UTRs of human *HDAC2/8* contain potential miR‐95‐5p binding sites (Figure 7A). Luciferase reporter assays with wild‐type or mutant versions of the 3ʹ‐UTRs of *HDAC2/8* were performed in the presence or absence of miR‐95‐5p overexpression. Transfection with miR‐95‐5p resulted in reduced luciferase activity, indicating reduced transcription of *HDAC2/8*, by binding to the wild‐type 3ʹ‐UTRs, while the mutant 3ʹ‐UTR sequences prevented the binding of miR‐95‐5p. This suggests that *HDAC2/8* are targets of miR‐95‐5p‐mediated repression (Figure 7B,C).

## 4 DISCUSSION

In this study, we analysed the expression profiles of miRNAs in exosomes isolated from the supernatants of OA and normal chondrocyte cultures. We found that 22 miRNAs were up‐regulated (fold change >2) and 29 miRNAs (including miR‐95‐5p) were down‐regulated (fold change <−2) in OA‐secreted exosomes compared to levels in normal chondrocyte‐secreted exosomes, as shown in Table S1. This study is the first to observe exosomal miR‐95‐5p‐mediated cartilage development and degradation via HDAC2/8. Our data showed that exosomal miR‐95‐5p was expressed in vitro. AC‐miR‐95‐5p‐Exos promoted cartilage development when hMSCs were induced to differentiate into chondrocytes. Furthermore, *HDAC2* and *HDAC8* were validated as targets of miR‐95‐5p with bioinformatics analysis, western blot assays and luciferase reporter assays. Moreover, in PHCs, treatment with AC‐anti‐miR‐95‐5p‐Exos enhanced HDAC2/8 expression and was associated with reduced expression of AcH3, aggrecan and Col2A1. In contrast, treatment with AC‐miR‐95‐5p‐Exos markedly suppressed HDAC2/8 production and was associated with increased levels of AcH3, aggrecan and Col2A1. These data suggest that exosomal miR‐95‐5p plays an active role in the regulation of HDAC2/8 during chondrogenesis and cartilage degeneration.

HDACs contain two families: the NAD^+^‐dependent protein deacetylases SIR2 family and the classical HDAC family. The SIR2 family, which include SIRT1‐7 members in mammals.^4^, ^32^, ^33^ In chondrocytes, SIRT1 was demonstrated to promote the SOX9 expression and also to enhance the acetylation activity of COL2A1 promoter regions.^34^ Classical HDACs contribute to the histones to wrap DNA more tightly to repress transcription via reducing the level of acetylation.^4^, ^12^ The role of the HDAC family in OA pathogenesis is complex. Class I HDACs (HDAC1, 2, 3 and 8) are important for cell proliferation and growth, gene replication and transcription,^9^ while class II HDACs (HDAC4, 5, 6, 7, 9 and 10) often utilize zinc (Zn^2+^) for their enzymatic activity and recruit class I HDACs for their catalytic function.^10^ Previous studies have shown that class I and class II HDACs play significant roles in the regulation of chondrocyte development and hypertrophic responses.^14^, ^16^, ^17^, ^18^, ^25^, ^26^, ^35^, ^36^ HDACs proteins cannot suppress all genes, but selectively repress a controlled set of cellular genes.^37^, ^38^ Previous study showed that knockout of a class I HDAC resulted in dramatic increases a wide‐ranging extracellular matrix‐related genes expression in *Caenorhabditis elegans*.^39^ In addition, the activity of HDAC1 and HDAC2 was responsible to the transcriptional repression of cartilage‐specific genes via the activation of NF‐κB signalling.^16^, ^40^ In this study, the knock‐down of *HDAC2* and *HDAC8* resulted in the up‐regulation of SOX9 and down‐regulation of RUNX2, respectively. Unexpectedly, miR‐95‐5p was found to target 2 class I HDACs, *HDAC2* and *HDAC8*.

There are few reports focusing on the link between miRNAs and HDACs during cartilage development and degradation. Previously, miR‐365, miR‐381, miR‐29b and miR‐222 were found to directly target *HDAC4* and to participate in chondrocyte differentiation, osteoblast differentiation and the pathogenesis of OA.^25^, ^27^, ^41^, ^42^ Here, we observed a negative correlation between the expression levels of exosomal miR‐95‐5p and HDAC2/8 in normal and OA tissues and exosomes. AC‐miR‐95‐5p‐Exos markedly promoted SOX9, aggrecan and COL2A1 expression during the early chondrogenic differentiation of hMSCs and maintained cartilage development. This was similar to the effects of miR‐95‐5p, which was shown to directly regulate the expression of *HDAC2*/*8* and enhance histone H3 acetylation.

There is increasing evidence that HDACi, such as trichostatin A (TSA) or belinostat (PXD101), suppress cartilage degradation and may be of potential therapeutic value for OA patients.^43^, ^44^, ^45^, ^46^ TSA promotes the acetylation of the *WNT5A* promoter, which in turn leads to the transcriptional suppression of COL2A1.^47^ TSA was also found to repress the expression of MMP1 and MMP13 in both IL‐1‐induced chondrocytes^9^ and a rabbit anterior cruciate ligament transection model of OA.^46^ Moreover, the underlying mechanism by which TSA affects cartilage development and degradation may involve histone modifications by HDACs. It has been reported that TSA induces histone H3/H4 acetylation and specifically increases H3/H4 acetylation levels near the *COL2A1* enhancer region.^9^ Interestingly, we noted that miR‐95‐5p functions, at least to some extent, in a manner similar to HDACi. Specifically, miR‐95‐5p inhibits HDAC2/8 expression, results in the hyperacetylation of histone H3 and increases the expression of cartilage matrix genes.

In conclusion, the results presented here demonstrate that the overexpression of exosomal miR‐95‐5p is essential during chondrogenesis to suppress *HDAC2/8* expression. To the best of our knowledge, this is the first study to demonstrate the regulation of class I HDACs by an exosomal miRNA during chondrogenesis. We also demonstrated that this mechanism plays a pivotal role in cartilage‐specific gene expression. Moreover, our data support a model in which exosomal miR‐95‐5p promotes cartilage formation through the modulation of histone modifications. These results not only provide new insights into chondrogenic regulation by class I HDACs and exosomal miRNAs, but also indicate that exosomal miR‐95‐5p may exert similar effects to HDACi and exhibit potential as a disease‐modifying osteoarthritis drug (DMOAD).

## CONFLICT OF INTEREST

The authors in this study declare that they have no conflict of interest.
